# Organoleptic characteristics of high‐protein snacks with novel and sustainable ingredients: Cricket flour and carob powder

**DOI:** 10.1002/fsn3.4392

**Published:** 2024-10-14

**Authors:** Reine Khalil, Zein Kallas, Montserrat Pujolà, Amira Haddarah

**Affiliations:** ^1^ Departament d'Enginyeria Agroalimentària i Biotecnologia (DEAB) Universitat Politècnica de Catalunya ‐ BarcelonaTech (UPC) Castelldefels Barcelona Spain; ^2^ Doctoral School of Sciences and Technology Lebanese University Hadath Lebanon; ^3^ Center for Agro‐Food Economics & Development (CREDA‐UPC‐IRTA) Castelldefels Barcelona Spain

**Keywords:** carob, cricket, flash profile, insect, nutrition claim, texture profile analysis

## Abstract

To fulfill consumer trends in sustainable and healthy food choices, this study explored the application of edible insects and carob powder as sustainable and nutritious ingredients in developing a high‐protein snack, known as a protein ball. Four formulations were developed and characterized in terms of moisture content, water activity, color, texture, microbial count, and nutritional profile. Finally, the sensory profile was determined using the flash profile method, and the developed product was compared to a commercial product. The effect of replacing the conventional protein source with cricket flour and cocoa with its sustainable alternative, carob, on the measured characteristics was determined. The results showed that cricket flour significantly decreased the lightness color values (from 40 to 30) on the internal surface of the protein ball. Texture remained largely unaffected initially; however, after 2 weeks of storage, cricket flour significantly decreased the hardness (from 15 to 12 N) and chewiness values (from 1.6 to 1.0 N mm). Moreover, cricket flour significantly increased the aerobic count (from 3–4 log to 5 log cfu g^−1^). The sensory space of the cricket samples was separated from the milk protein samples, mainly related to flavor attributes, while the commercial sample was distinguished by dryness and sweetness. In general, carob powder did not affect the measured parameters compared to cocoa. This study demonstrated the suitability of utilizing cricket flour and carob powder in a high‐protein snack without substantially compromising the product's organoleptic properties. Future research could investigate the consumer acceptability of the product.

## Graphical abstract

The protein balls were characterized in terms of their organoleptic properties including color, texture, and sensory profiling, along with nutritional composition, microbiological count, moisture content, and water activity. Our results demonstrated a potential application of cricket flour and carob powder as sustainable ingredients in high‐protein snacks without substantially compromising its properties.

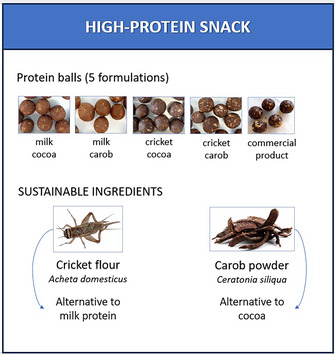

## 1 INTRODUCTION

High protein and environmental sustainability are two major trends currently shaping the food industry (Castro‐Delgado et al., 2020). Protein is the main macronutrient in our body that supports weight management, promotes satiety, improves body composition, and maintains and increases muscle mass (Mirazimi et al., 2022). This is particularly important for athletes and the elderly. The recommended daily protein intake for the European population is 0.83 g kg^−1^ body weight, while athletes require 1.4–3.0 g kg^−1^ body weight depending on their type of activity and physiological state. Therefore, consumers who engage in sports are an ideal target for high‐protein products (Grdeń & Sołowiej, 2022). For example, protein shakes, ready‐to‐mix powders, and protein bars are popular products among athletes and regular consumers alike. They come in a wide range of flavors and feature various health and nutrition claims (Harwood & Drake, 2019; Kooh et al., 2020). According to regulation EC No. 1924/2006, a product may be considered high in protein if at least 20% of its energy value is provided by protein.

Among the novel foods under the EU regulation 2015/2283 (EC, 2015), insects for human consumption have been extensively explored by researchers and industry professionals. Edible insects are a complete source of proteins, containing all essential amino acids (Chamoun et al., 2023; Orkusz, 2021). The house cricket protein, in particular, has a high level of leucine, a key amino acid for muscle growth, which meets the required amount per meal, as reported by Grdeń and Sołowiej (2022). Udomsil et al. (2019) also confirmed that house crickets (*Acheta domesticus*) are a rich source of protein, comprising 70% of their dry matter. It also contained all essential amino acids in quantities similar to or higher than the levels recommended by FAO (Turck et al., 2021). Insect protein digestibility is generally considered high (Biró et al., 2019; Detilleux et al., 2021; Ronoh et al., 2024), estimated to be between 75% and 98% (Osimani et al., 2017; Thrastardottir et al., 2021) and 83.9% for *Acheta domesticus* (Magara et al., 2021). Although it is slightly less digestible than beef and eggs, edible insect protein is more digestible than plant proteins, as noted by Gnana Moorthy Eswaran et al. (2023) and Ochieng et al. (2023). In addition to their high‐quality protein, edible insects can provide a satisfactory level of minerals. House crickets, for example, are rich in calcium (Gere et al., 2019), zinc (Payne et al., 2016), magnesium, and iron (Udomsil et al., 2019) which are essential for muscle function and bone tissue maintenance (Thomas et al., 2016).

Introducing edible insects, such as crickets, into human diets can promote sustainability compared to conventional protein sources (Gurdian et al., 2021). They serve as a complementary food source in developed countries (Caparros Megido et al., 2014; Sogari et al., 2017) that can be incorporated into snacks, beverages, bakery products, and meat emulsion products (sausages, burgers, etc.) (Acosta‐Estrada et al., 2021). The sustainability of edible insects is mainly supported by their high feed conversion efficiency (Naranjo‐Guevara et al., 2021; Rodríguez‐Miranda et al., 2019) and their ability to convert low‐value organic by‐products into high‐value proteins (Mustapa & Kallas, 2023; van Huis & Oonincx, 2017). In addition, compared to the production of conventional animal proteins, they emit less greenhouse gases and ammonia, consume less water, and use less space (Kasza et al., 2023). Life‐cycle assessments can determine more precisely the environmental impact of edible insect production from a holistic approach. For example, a life‐cycle assessment of cricket farming in Thailand was conducted by Halloran et al. (2017) comparing it to broiler production and found that the environmental impact of the former was lower in almost all the impact categories. More relevant to our case, Smetana et al. (2016) compared the production of insect protein powder from black soldier fly larvae (*Hermetia illucens*) to that of whey protein powder, a popular sport dietary supplement. They found that insect powder was at least twice more sustainable.

The use of insect flour as a protein‐rich ingredient has been explored in different products throughout the literature. Its effect on different technological, physicochemical, microbiological, and sensory properties of the product has been studied. The most commonly evaluated product was bread explored by de Oliveira et al. (2017), da Rosa Machado and Thys (2019), González et al. (2019), Haber et al. (2019), Osimani et al. (2018), Roncolini et al. (2019), and Bartkiene et al. (2022). Insect flour has also been explored in the development of cookies (Ochieng et al., 2023; Sriprablom et al., 2022), biscuits (Ayensu et al., 2019; Ronoh et al., 2024), meat emulsion (Ho et al., 2022; Kim et al., 2016, 2017), pasta (Biró et al., 2019; Ho et al., 2022), brownie (Ho et al., 2022), imitation mozzarella cheese (Chailangka et al., 2023), fermented seasoning sauce (Cho et al., 2018), and extruded snacks (García‐Segovia et al., 2020).

In this study, protein balls were developed considering them a convenient high‐protein snack that can be consumed after a workout to increase energy intake and support muscle growth and recovery. Ardoin and Prinyawiwatkul (2020) found that protein/energy bars were the most acceptable product by consumers to be incorporated with insects. Protein balls are similar to bars in composition but are of a different shape. The product was formulated by introducing two sustainable ingredients, cricket flour and carob powder. They are alternatives to two ingredients that are most commonly found in sports products in the marketplace, milk protein (Liu et al., 2021) and cocoa powder (Campbell et al., 2016; Harwood & Drake, 2019), respectively. Cocoa also helps in masking the color of cricket flour. Hence, four formulations were studied alternating the factor of the main protein source (milk protein isolate and cricket flour) and the factor of the flavoring ingredient (cocoa and carob). The control sample was the formulation based on milk protein and cocoa powder, the conventional ingredients in sports products. On the other hand, the insect species *Acheta domesticus* was explored in this study considering that it is the most widely consumed insect (Boukid et al., 2022; Sipponen et al., 2018) and one of the most accepted edible insects by Western consumers alongside mealworms and grasshoppers (Gurdian et al., 2021). Additionally, carob powder was selected since it is a more sustainable alternative to cocoa. It has a similar flavor and appearance to chocolate (Yousif & Alghzawi, 2000). The carob tree (*Ceratonia siliqua*) is native to the Mediterranean region and plays a role in the reforestation of arid and degraded areas, and in combating forest fires (Haddarah et al., 2014).

Furthermore, this study explored a recent sensory descriptive technique, the flash profile (FP) method. The most common technique used for descriptive sensory analysis is quantitative descriptive analysis (QDA). However, it incurs long hours of training for the assessors to translate their sensory perceptions using the same vocabulary which can be overwhelming (Delarue & Sieffermann, 2004). Rapid methods have been developed that do not require the training of assessors such as the FP method. These methods are not a substitute for conventional QDA but can provide valuable and reliable sensory profiles in a relatively short time (Bredie et al., 2018; Delarue & Sieffermann, 2004). Flash profile is a variant of Free‐Choice Profiling that is based on ranking instead of rating (Liu et al., 2018). In this method, the attributes used to describe the product are free of choice where each assessor generates his/her individual descriptive profile of the product. Then, a consensus configuration is obtained by Generalized Procrustes Analysis (GPA) which applies transformations (scaling, translations, and rotations) to the individual data matrices of each assessor (XLSTAT, 2017).

The FP method depends on the involvement of panelists with sensory analysis experience who are able to translate their sensory perceptions into meaningful descriptors avoiding hedonic terms (Rodríguez‐Noriega et al., 2021). The number of panel members generally used in studies is 6–12 trained or semi‐trained members (Liu et al., 2018; Rodríguez‐Noriega et al., 2021). The maximum number of samples to be evaluated should not exceed 10–12 samples according to Rodríguez‐Noriega et al. (2021). For example, the number of samples was four in Mamede and Benassi (2016), Montanuci et al. (2015), and Rodríguez‐Noriega et al. (2021); five in Delarue and Sieffermann (2004); six in Delarue and Sieffermann (2004) (for another product) and Price et al. (2019); and nine in Liu et al. (2018) and Dehlholm et al. (2012).

Other studies have used the FP method to evaluate the sensory characteristics of different products. For example, Price et al. (2019) applied FP on a cross‐cultural study to profile six samples of honey. In Rodríguez‐Noriega et al. (2021), it was applied on four flour tortilla samples with 10 trained panelists. While Liu et al. (2018) profiled nine wine samples comparing different rapid descriptive sensory methods.

Therefore, the objective of this study was to develop a high‐protein snack exploring two sustainable ingredients, cricket flour and carob powder. The organoleptic properties (color and texture) were evaluated at two different times of storage along with moisture content, water activity, and microbiological assessment. The sensory properties of the product using the FP method were evaluated. Our formulations were compared to a commercially available protein ball. To the best of our knowledge, this is the first study to evaluate protein balls formulated with cricket flour and carob powder, and its organoleptic characteristics. Chow et al. (2021) studied the hedonic response of children to a similar product under a different name “oatmeal balls” and using a different type of insect.

## 2 MATERIALS AND METHODS

### 2.1 Materials

The ingredients such as peanut butter (100% natural), rolled oats, honey, cocoa powder (100% pure), sunflower oil, and salt were purchased from a local supermarket. The carob powder (BIOCOP, Productos Biológicos, S.A., Barcelona, Spain) was purchased from an online store. The cricket flour (100% *Acheta domesticus*) was purchased from Origen Farms, S.L., Albacete, Spain. The milk protein isolate (90% protein: 80% casein + 20% whey) was purchased from the HSN's online store (Harrison Sport Nutrition S.L., Granada, Spain). The sodium alginate of Sigma Aldrich brand was purchased from Merck S.L, Barcelona, Spain. The commercial protein balls (peanut‐cocoa flavor) were purchased from Foodspring GmbH, Berlin, Germany.

### 2.2 Sample preparation

The protein balls were prepared in the sensory analysis laboratory of the Barcelona School of Agri‐food and Biosystems Engineering (EEABB) at UPC‐BarcelonaTech. Preliminary tests were done to determine the optimal recipe while exploring different ingredients. The amount of cricket flour or milk protein in the formulation was determined based on the “high protein” claim of the regulation 1924/2006 (EC, 2006), which requires that at least 20% of the energy value of the product is provided by protein. According to the Federal Agency for the Safety of the Food Chain (FASFC, 2014), the daily intake of 45 g of freeze‐dried insects containing, on average, 6% chitin is not a health concern. In our product, the serving size is 5 balls or 40 g, containing approximately 7% dried cricket flour, as indicated in Table 1. According to Kim et al. (2017), Sipponen et al. (2018), and Turck et al. (2021), chitin is estimated to range from 4% to 8% on a dry weight basis in adult house crickets. Therefore, chitin intake from our product is well below the established limit and poses no health risks.

**TABLE 1:** Formulation of the four protein balls.

Ingredients	Milk protein cocoa	Milk protein carob	Cricket cocoa	Cricket carob
Peanut butter (g)	30.52	30.52	29.85	30.37
Water (g)	26.16	26.16	24.45	24.30
Oats (g)	19.77	19.77	19.33	19.67
Honey (g)	11.63	10.47	12.51	10.41
Cricket powder (g)	–	–	7.39	7.52
Milk protein (g)	7.56	7.56	1.99	2.02
Cocoa or carob (g)	2.03	3.20	1.99	3.18
Sunflower oil (g)	1.74	1.74	1.71	1.74
Salt (g)	0.58	0.58	0.57	0.58
Alginate (g)	–	–	0.20	0.20

*Note*: Ingredients are expressed per 100 g ball. “–”: not included in the formulation.

**FIGURE 1:**
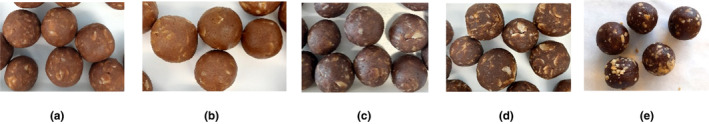
Protein ball formulations: (a) milk protein cocoa; (b) milk protein carob; (c) cricket cocoa; (d) cricket carob; (e) commercial sample.

To prepare the protein balls, first, rolled oats were toasted in the oven at 160°C for 3 min. All the ingredients were weighed according to the formulation in Table 1. Next, the honey and water were mixed by hand until the honey was dissolved in the water. The dry ingredients (cricket flour and/or milk protein, cocoa or carob powder, salt, and/or alginate) were sifted. The ingredients were mixed with Kenwood Chef stand mixer (700 w, 4.6 L bowl) starting with the dry ingredients. Second, the honey and water mixture were added to the bowl through the opening while it was being mixed with the dry ingredients. Third, the peanut butter was added to the bowl and mixed with the rest of the ingredients while adding the oil through the opening. Finally, the oat was added to the bowl and kneaded by hand. After the mix was prepared, it was divided into smaller pieces of equal size (8 g) using a mold, and each piece was rolled by hand into a ball. The prepared protein balls and the commercial sample are shown in Figure 1.

The balls were packed in polypropylene trays using Easybox FP thermosealer (ILPRA Systems España S.L, Barcelona, Spain). The thermosealant films were provided by Tecnopack srl (Mortara, Italy) having a thickness of 84 μm, water vapor permeability of <10 g/m^2^/24 h/bar, and oxygen permeability of <1 mL/m^2^/24 h/bar. The trays were provided by ATS Packaging srl (Mirano VE, Italy). They were stored in a refrigerator (2°C ± 1°C) upon their further usage. The product was stored in a refrigerator to control microbial growth and extend its shelf life considering its high water activity as shown in the results.

### 2.3 Moisture content

The ground samples were oven‐dried using a Digitheat natural convection oven at 102°C ± 2°C for 24 h according to the method described in AOAC (1995) and Maleki et al. (2023). They were then cooled in a desiccator and weighed to determine moisture content. Six replicates were evaluated per treatment after 1 day and 2 weeks of refrigerated storage.

### 2.4 Water activity

Water activity was measured using the LabMaster‐aw neo machine (Novasina AG., Lachen, Switzerland) at 25°C. The measurement was determined by establishing a humidity equilibrium between the free, available water in the sample and the humidity of the air above the sample surface according to the ISO 18787: 2017 standard requirements. A round slice of the ball was spread over the dish of the apparatus. Six replicates were evaluated per treatment after 1 day and 2 weeks of refrigerated storage.

### 2.5 Nutritional composition

The approximate nutritional composition (fat, protein, carbohydrates, sugar, and fiber) and calorie content of the formulations per 100 g was determined by calculation considering the nutritional and calorie content of each ingredient from the specifications provided by the manufacturers.

### 2.6 Color measurement

The color parameters L*, a*, and b* were measured using the colorimeter Chroma Meter CR‐400 (Konica Minolta Sensing, Inc., Tokyo, Japan). The colorimeter was calibrated using a white ceramic standard. The chroma was calculated as $C^{*}=\sqrt{a^{{*}^{2}}+b^{{*}^{2}}}$, hue angle as $H^{o}=\tan^{-1}\left(\frac{b^{*}}{a^{*}}\right)$, and mean color difference of the products between initial time and 2 weeks of storage as $\Delta E=\sqrt{{\left(\Delta {\bar{L}}^{*}\right)}^{2}+{\left(\Delta {\bar{a}}^{*}\right)}^{2}+{\left(\Delta {\bar{b}}^{*}\right)}^{2}}$. Lightness indicates the proportion of the light reflected, chroma indicates colorfulness relative to the brightness of its surroundings, and hue indicates the attribute described by color names (red, green, purple, etc.) (Benítez et al., 2015; MacDougall, 2001; McGuire, 1992).

According to Mokrzycki and Tatol (Mokrzycki & Tatol, 2011), Δ*E* can be interpreted as follows:

- 0 < Δ*E* < 1: the difference is not noticed by observers
- 1 < Δ*E* < 2: the difference is only noticed by experienced observers
- 2 < Δ*E* < 3.5: the difference is also noticed by unexperienced observers
- 3.5 < Δ*E* < 5: a clear color difference is noticed
- 5 < Δ*E*: two different colors are noticed by observers

The balls were cut into round slices to measure the color of the internal surfaces. Six replicates were evaluated per treatment. The color was evaluated after 1 day and 2 weeks of refrigerated storage.

### 2.7 Texture profile analysis (TPA)

Texture profile analysis (TPA) was performed at 20°C by the Texture Analyzer (TA.XTplus, Stable Microsystems, Godalming, UK) with a load cell of 5 kg. Double‐cycle compression tests were performed at a pre‐test speed of 1 mm s^−1^, a test and post‐test speed of 5 mm s^−1^, a distance of 10 mm, and a pause time between compression cycles of 5 s using a probe with a diameter of 75 mm. The textural properties measured were hardness, springiness, cohesiveness, and chewiness. Hardness indicates the peak force during the first compression cycle, cohesiveness indicates how well the product withstands a second deformation relative to its resistance under the first deformation, springiness indicates how well a product springs back after the first compression, and chewiness is calculated as the product of hardness times cohesiveness times springiness (da Rosa Machado & Thys, 2019; Johnson, 2015; Mochizuki, 2001). Eighteen replicates were evaluated per treatment without slicing or cutting the protein ball, after 1 day and 2 weeks of refrigeration.

### 2.8 Microbiological analysis

The samples were tested for mesophilic aerobic microorganisms, yeasts, and molds after 1 day and 2 weeks of refrigeration according to the methods described in AOAC (1995). The analyses were carried out in an accredited external laboratory (Silliker Mérieux NutriSciences, Barcelona, Spain).

### 2.9 Descriptive sensory analysis by a trained panel

The descriptive sensory evaluation of the products was done using the FP method (Bredie et al., 2018; Dairou & Sieffermann, 2002) with some modifications. Eleven trained sensory panelists that belong to the Miquel Agustí Foundation (FMA) evaluated the four different formulations in addition to the commercial product. The sensory evaluation involved two sessions. Each session starts with a briefing about the evaluation process and the samples.

The first session consisted of generating the attributes that best describe the differences among the samples. To assist the panel, seven attributes were pre‐selected based on a pre‐test carried out by three different panelists and were listed in the score sheet. The panel was free to generate more attributes or exclude the existing ones. In addition, they were handed a sheet of descriptors that may be associated with the product along with their definitions. In the same session, the panel was asked to rank the samples in ascending order from low intensity to high intensity based on each attribute in their definitive list. Ties were allowed when no difference was perceived between two or more samples. Delarue (2014) recommends joining these two steps of attribute elicitation and ranking into one step. He has found that comparing the attributes generated by each panelist with the pooled list of the attributes generated by all the panel, as the method suggests, has limited added value where the panelists usually hold on to their original list.

The second session consisted of a repetition of the ranking. Codes were independent for each session and samples were presented in a randomized order. The scoring sheets are available in Appendix S1. The sensory evaluation took place in the sensory analysis laboratory of the Barcelona School of Agri‐food and Biosystems Engineering (EEABB) at UPC‐BarcelonaTech and was carried out in the Spanish language. Ethical approval was obtained from the ethics committee of the Center for Agro‐food Economics and Development (CREDA) and participants gave informed consent.

For data analysis, the attributes that do not differentiate the samples were eliminated as recommended by Dairou & Sieffermann (2002). Moreover, the reliability of the tasters was determined by testing the Spearman correlation coefficient between the replicates of each attribute (AENOR, 2017; Price et al., 2019).

The safety of the products was confirmed prior to conducting the sensory analysis. Cricket flour was tested for Bacillus cereus (< 1 log CFU g^−1^), Enterobacteria (< 1 log CFU g^−1^), and coagulase‐positive Staphylococci (< 1 log CFU g^−1^). All five protein ball formulations were tested for total aerobic count, yeasts, and molds, as described in the previous Section 2.8. All results were within the safe limits. Panelists were informed of the presence of cricket ingredients in some of the protein ball samples. Furthermore, pregnant or lactating women, individuals with allergies to specific ingredients, those below 18 years of age, those with any special health condition, vegans, or vegetarians were not allowed to participate in the study.

### 2.10 Statistical analysis

The *T*‐test (two‐sided) was used to compare the means of the two groups. Analysis of variance (ANOVA) was used to determine if there is a significant difference between means of more than two groups followed by Tukey's comparison test. When the homogeneity of variance assumption was violated, Welch's *F* test was used followed by the Games–Howell post hoc test to compare and rank the differences. Statistical significance was determined at a 5% level. The software used for these tests was SPSS Statistics v. 29.0 (IBM Corp., New York, USA). In the case of FP, data were analyzed by Generalized Procrustes Analysis using XLSTAT software (version 2023.1.1, Addinsoft, New York, USA) and was applied to the mean rank data of the two sessions. The discrimination of the attributes for each panelist was tested by the Kruskal–Wallis test. The repeatability of the panelists between the two sessions was tested by the Spearman correlation test.

## 3 RESULTS

### 3.1 Moisture content and water activity

The moisture content and water activity of the different protein ball formulations are shown in Table 2. Initially, the moisture content and water activity values of the milk protein samples were significantly higher (*p* < .05) than those of the cricket samples. Additionally, within the same protein source, the cocoa samples exhibited higher moisture content compared to the carob samples. After 2 weeks of refrigerated storage, the moisture content was generally similar across all formulations. The water activity of the milk protein formulations remained significantly higher (*p* < .05) than the cricket formulations. Regarding the effect of time, changes in moisture content varied among the samples. On the other hand, the water activity significantly increased after 2 weeks of refrigeration for all samples.

**TABLE 2:** Moisture content and water activity of the different protein ball formulations after 1 day and 2 weeks of refrigerated storage (2°C ± 1°C).

Formulation	Moisture (g 100 g^−1^)		Water activity	
	Initial	2 weeks	Initial	2 weeks
Milk protein cocoa	31.06 ± 0.24^Ax^	30.48 ± 0.33^Ay^	0.907 ± 0.002^By^	0.912 ± 0.001^Bx^
Milk protein carob	30.37 ± 0.12^By^	30.47 ± 0.20^Ax^	0.909 ± 0.001^Ay^	0.916 ± 0.001^Ax^
Cricket cocoa	30.05 ± 0.07^Cy^	30.39 ± 0.24^Ax^	0.894 ± 0.001^Cy^	0.897 ± 0.001^Cx^
Cricket carob	29.59 ± 0.15^Dx^	29.08 ± 0.22^By^	0.895 ± 0.001^Cy^	0.899 ± 0.002^Cx^

Values are mean ± standard deviation. Effect of formulation: Different letters (A, B, C, D) in the same column indicate statistical difference at 5%. Effect of time: Different letters (*x*, *y*) in the same row of the same parameter and same formulation indicate statistical difference at 5%.

### 3.2 Nutritional composition

Table 3 presents the nutritional data for the different protein ball formulations. The values for most nutrients were largely consistent across formulations, except for the fat and calorie content, which were higher in the cricket formulations. The cricket carob sample had the highest fiber content, and the cricket cocoa sample had the highest sugar content. The protein content in all samples was sufficient to achieve the “high protein” claim which requires that at least 20% of the energy value is provided by protein, as stipulated by regulation EC No. 1924/2006.

**TABLE 3:** Nutritional data of the different protein ball formulations.

Formulation	Fat (g)	Protein (g)	Carbohydrates (g)	Sugar (g)	Fiber (g)	Calories (kcal)
Milk protein cocoa	18.73	19.14	24.51	10.65	5.40	354.38
Milk protein carob	18.53	18.83	24.56	10.88	6.13	352.71
Cricket cocoa	19.61	19.00	25.57	11.27	5.73	365.82
Cricket carob	19.74	19.01	25.10	10.78	6.56	366.65

*Note*: Nutritional data are expressed per 100 g ball.

### 3.3 Color

**FIGURE 2:**
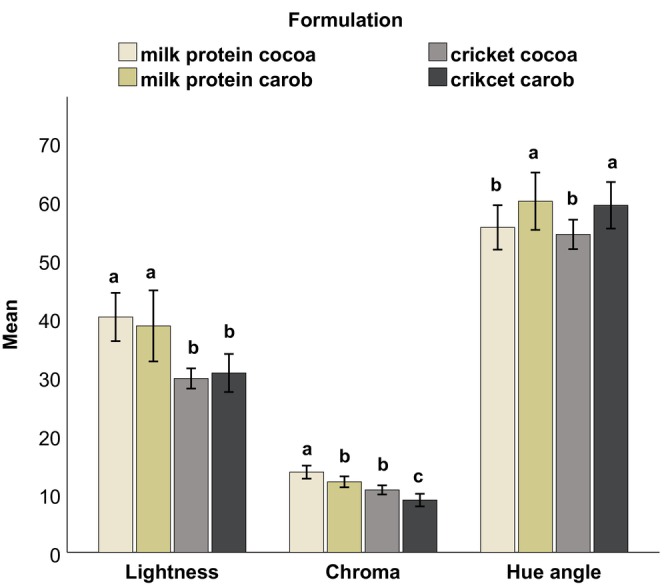
Lightness, chroma, and Hue angle of the different formulations at initial time from the internal side of the protein ball. Error bars: ±1 SD. Different letters indicate statistical difference at 5%.

**FIGURE 3:**
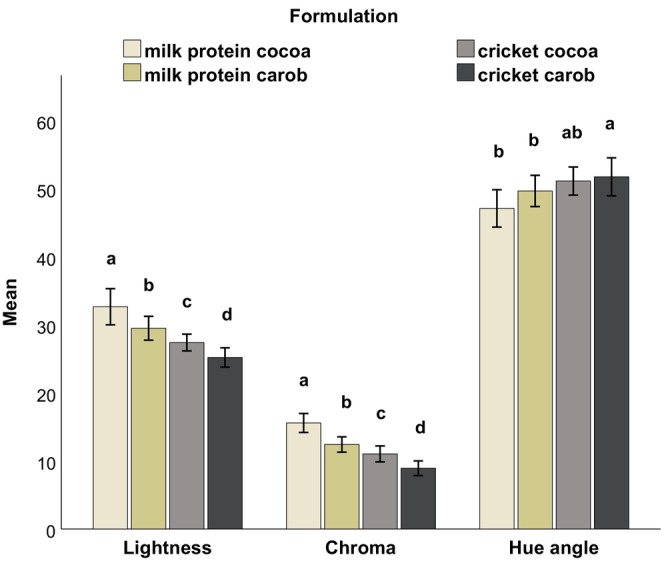
Lightness, chroma, and Hue angle of the different formulations at initial time from the external side of the protein ball. Error bars: ±1 SD. Different letters indicate statistical difference at 5%.

The results for the color parameters lightness (L*), chroma (C*), and Hue angle (*H* ^*o*^) are illustrated in Figures 2 and 3, depicting both the internal and external sides of the protein balls at initial time. The color of the milk formulations was lighter and brighter than the cricket formulations. The L* values of the milk formulations (40.18 and 39.62 internal, 32.66 and 29.50 external) were significantly higher (*p* < .05) than those of the cricket formulations (29.68 and 30.62 internal, 27.40 and 25.20 external) on both sides. Similarly, the C* values of the milk formulations (13.70 and 11.42 internal, 15.58 and 12.42 external) tended to be higher than those of the cricket formulations (10.65 and 8.92 internal, 11.04 and 8.93 external).

Concerning the effect of the replacement of cocoa with carob, it resulted in a significant increase (*p* < .05) in the Hue angle on the internal side of the balls (59.95° and 59.25°) compared to that of the cocoa formulations (55.47° and 54.28°). Additionally, it significantly decreased the C* values in the formulations of the same protein source for both internal and external sides.

The color difference (Δ*E*) of the different treatments between 1 day and 2 weeks of refrigerated storage was determined from the average values of L*, a*, and b*. The cricket cocoa formulation exhibited the highest level of color change after 2 weeks of storage, both internally (2.97) and externally (2.64). In contrast, the other formulations had lower Δ*E* values. The Δ*E* values were 1.30 for milk cocoa, 2.18 for milk carob, and 0.93 for cricket carob internally. Externally, the values were 0.54 for milk cocoa, 1.13 for milk carob, and 1.00 for cricket carob. Notably, the internal and external Δ*E* values of each cricket formulation were more similar to each other, while for each milk protein formulation, the Δ*E* values of the internal side of the balls were higher than the external side.

### 3.4 Texture profile analysis (TPA)

Table 4 presents the TPA of the different formulations in terms of hardness, springiness, cohesiveness, and chewiness; measured after 1 day and 2 weeks of refrigerated storage.

**TABLE 4:** Texture profile analysis (TPA) of the different protein ball formulations after 1 day and 2 weeks of refrigerated storage (2°C ± 1°C).

Formulation	Hardness (N)		Springiness (mm)		Cohesiveness (−)		Chewiness (N.Mm)	
	Initial	2 weeks	Initial	2 weeks	Initial	2 weeks	Initial	2 weeks
Milk cocoa	12.90 ± 1.27^Ax^	15.59 ± 1.95^Ax^	0.32 ± 0.02^Ay^	0.37 ± 0.03^Ax^	0.26 ± 0.01^Ay^	0.28 ± 0.01^Ax^	1.12 ± 0.14^Ay^	1.61 ± 0.18^Ax^
Milk carob	11.18 ± 1.29^Bx^	14.86 ± 1.08^Ax^	0.28 ± 0.03^Cy^	0.36 ± 0.02^Ax^	0.26 ± 0.01^Ay^	0.28 ± 0.01^ABx^	0.79 ± 0.10^BCy^	1.57 ± 0.13^Ax^
Cricket cocoa	12.23 ± 2.62^ABx^	11.89 ± 1.74^Bx^	0.29 ± 0.04^BCy^	0.31 ± 0.03^Bx^	0.25 ± 0.03^Ax^	0.27 ± 0.01^Bx^	0.70 ± 0.17^Cy^	0.95 ± 0.13^Bx^
Cricket carob	12.20 ± 1.80^ABx^	12.78 ± 1.72^Bx^	0.32 ± 0.03^ABy^	0.35 ± 0.04^Ax^	0.23 ± 0.01^Bx^	0.24 ± 0.01^Cx^	0.84 ± 0.15^By^	1.00 ± 0.18^Bx^

*Note*: Values are mean ± standard deviation. Effect of formulation: Different letters (A, B, C, and D) in the same column indicate statistical difference at 5%. Effect of time: Different letters (*x*, *y*) in the same row of the same parameter and same formulation indicate statistical difference at 5%.

Initially, the hardness and springiness values of the cricket and milk formulations were generally similar. However, the cohesiveness of the cricket carob formulation was significantly lower (*p* < .05) than that of the other formulations. Additionally, the milk cocoa formulation had the highest chewiness value.

After 2 weeks of storage, the differences between the milk protein and cricket formulations in terms of hardness and chewiness were more apparent. The hardness and chewiness of the milk protein formulations were significantly higher (*p* < .05) than those of the cricket formulations. However, the springiness and cohesiveness parameters were relatively similar across all formulations.

As for the effect of storage time, all texture parameters increased significantly (*p* < .05) after 2 weeks of storage for both milk protein samples. As for the cricket samples, only the springiness and chewiness values increased significantly while the hardness and cohesiveness values were maintained. These findings suggest that storage time affects the texture of milk protein samples more than cricket samples.

Concerning the effect of cocoa and carob flavors on texture and comparing the two different milk protein formulations, we can see that the milk cocoa formulation exhibited a significantly higher hardness, springiness, and chewiness compared to the milk carob formulation after 1 day of refrigeration. Nevertheless, the differences were not observable after 2 weeks of refrigerated storage. Comparing the two cricket formulations, there were no significant differences in hardness between the cocoa and carob formulations at initial time, and in hardness and chewiness after 2 weeks of refrigerated storage.

### 3.5 Microbiological analysis

**FIGURE 4:**
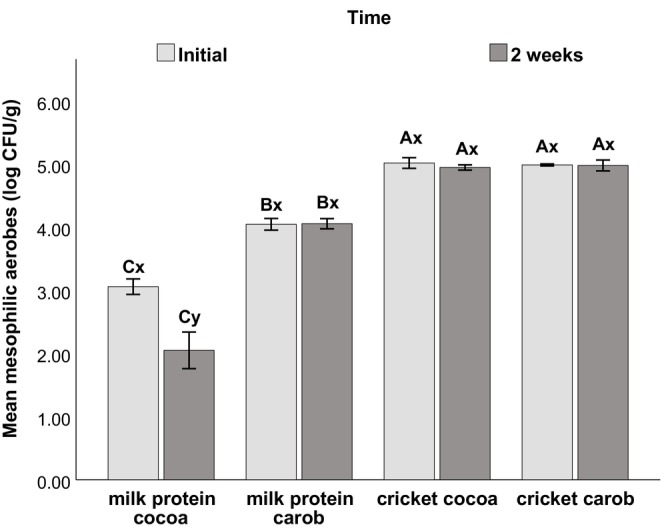
Mesophilic aerobes count of the different protein ball formulations at initial time and after 2 weeks of refrigerated storage (2°C ± 1°C). Error bars: ±1 SD. Effect of formulation: Different letters (A,B,C,D) indicate statistical difference at 5% between formulations at a given time. Effect of time: Different letters (*x*, *y*) indicate statistical difference at 5% across time for a given formulation.

**FIGURE 5:**
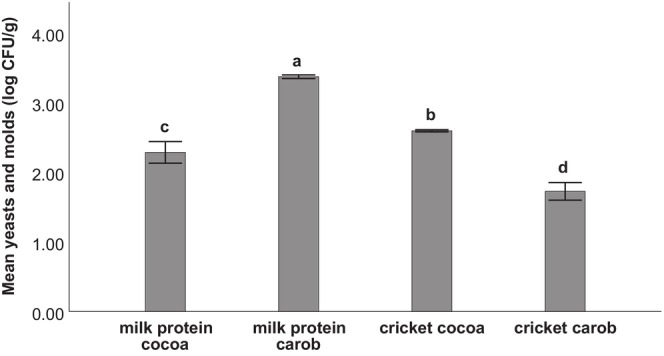
Yeasts and molds count of the different protein ball formulations after 2 weeks of refrigerated storage (2°C ± 1°C). Error bars: ±1 SD. Different letters indicate statistical difference at 5%.

The results of the mesophilic aerobes and yeasts and mold count of the different formulations are shown in Figures 4 and 5. Replacing milk protein powder with cricket flour significantly increased the mesophilic aerobes count at initial time and after 2 weeks of storage. Additionally, the mesophilic aerobes count of the milk carob formulation was significantly higher (*p* < .05) than that of milk cocoa. The values were maintained after 2 weeks of refrigeration without a significant difference except for the milk cocoa formulation which showed a significant decrease. As for the count of yeasts and molds, the milk carob formulation had the highest count.

### 3.6 Commercial product description

Considering the importance of sensory analysis in assessing market positioning, a commercial product was included along with the experimental samples. The commercial product was packed in a modified atmosphere, stored at ambient temperature, did not contain added water, and was produced on a different date than the experimental samples. Considering these differences, the commercial product was not included in the previous analyses. The values of the measured parameters of the commercial product are provided in the Appendix S3, which took place on the same day of opening the packaging.

In general, the moisture content and water activity values of the commercial product were lower than those of the experimental samples, while the sugar content and energy values were higher. The mean color difference, Δ*E*, was calculated by comparing the color of the experimental samples with that of the commercial product. Accordingly, the results showed that the color of the commercial product was closest to that of the cricket cocoa sample internally and that of the milk carob sample externally. The chewiness and hardness values were considerably higher than those of the experimental samples. Finally, the mesophilic aerobes and yeasts and molds counts of the commercial product were lower.

### 3.7 Descriptive sensory analysis

**FIGURE 6:**
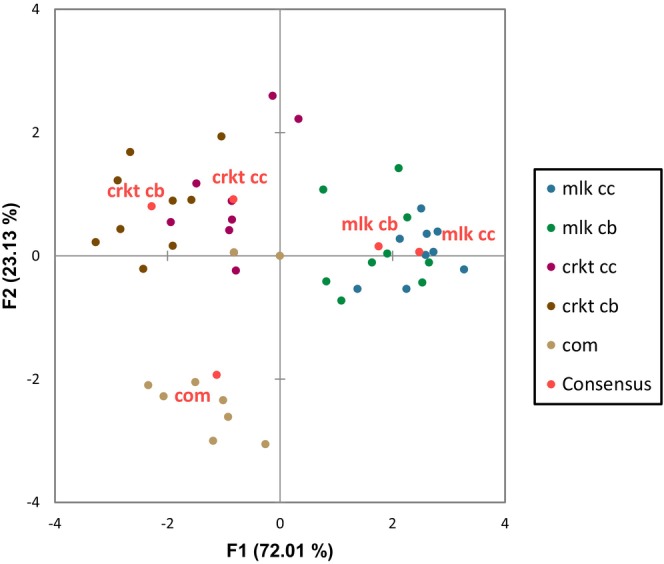
Sensory map of protein ball samples from GPA of flash profile data. (mlk cc: Milk cocoa; mlk cb: Milk carob; crkt cc: Cricket cocoa; crkt cb: Cricket carob; com: Commercial)

**FIGURE 7:**
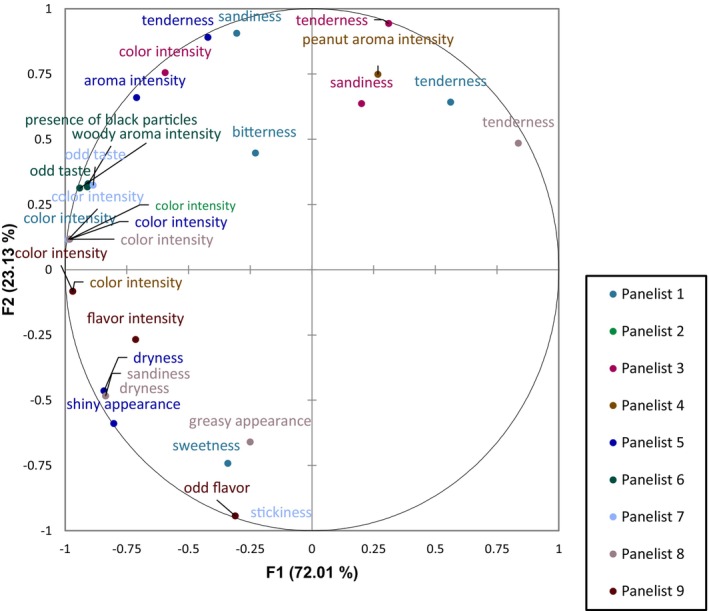
Loading plots for attributes of each panelist.

The panel generated 18 unique attributes other than the seven suggested attributes, ranging between seven and 11 attributes for each panelist. The attributes for each panelist that did not discriminate (*p* value of Kruskal–Wallis ≥ .1) the samples were eliminated. The significance was determined at 10% instead of 5% to reduce the number of eliminated attributes. Additionally, the attributes of each panelist that were not well correlated (Spearman correlation coefficient < .6) between the first and second sessions were also eliminated. The results of the filtering tests are available in Appendix S2. Consequently, two panelists were eliminated, and 15 different attributes were retained in the analysis ranging between one and five attributes for each panelist. The residuals by sample after transformations ranged from 4.35 for the milk cocoa sample to 17.64 for the commercial sample. The residuals by panelists after transformations ranged from 2.22 to 9.49. The consensus index (*R*_C_) value obtained was 0.371. The first component of the GPA consensus represented 72.01% of the variance and the second 23.13% with a cumulative variability of 95.14%. The sensory mapping of the five samples is illustrated in Figure 6 and the loading plots for the attributes are shown in Figure 7.

The results showed three groups that are clearly separated by the two components (Figure 6): milk protein, cricket, and commercial samples. The milk protein samples tended toward the attribute tenderness and away from the attributes of color intensity, odd taste, dryness, and sandiness along the first component. Since the consensus of both milk samples is almost positioned along the center of the second component, they did not tend toward any attribute related to this component. As for the cricket samples, regarding the first component, they could be associated with color intensity, odd taste, flavor intensity, aroma intensity, and woody aroma. While along F2, they can be associated with tenderness, peanut aroma, and sandiness. Finally, the commercial sample tended toward color intensity, dryness, odd taste, flavor intensity, and shiny appearance along F1. Along F2, it tended away from tenderness and toward sweetness, greasy appearance, and stickiness. Corresponding to the cocoa and carob samples of the same protein source, they are positioned in the same area of the map and therefore, share similar attributes.

## 4 DISCUSSION

### 4.1 Moisture content and water activity

Protein balls were developed as a convenient, high‐protein post‐workout snack. The cricket formulations included alginate and a slightly lower water content due to the lower water‐holding capacity of cricket flour compared to milk protein powder. This resulted in a lower moisture content and water activity of the cricket formulations at initial time (Table 2). Cricket flour has a lower protein content (69%) than milk protein powder (90%), which reduces its water absorption capacity. Additionally, the amino acid composition between different proteins can impact water absorption capacity (González et al., 2019). Food products often lose water when stored in the refrigerator (Hoffmann et al., 2023; Laguerre et al., 2010). The thermosealant films used for storing the samples have a relatively low water vapor permeability. Therefore, the moisture loss in our samples during storage was not evident. The increase in water activity of the samples during storage may be attributed to moisture migration, resulting in more free water as networks form between other components.

Compared to other studies, the moisture content and water activity values in our samples were found to be higher. For instance, the vegan bonbons developed by Mihaylova et al. (2022) had a moisture content range of 5.0–7.5 g 100 g^−1^, with no added water in the formulation. As for the water activity values, it was in the range of 0.468–0.559 in Mihaylova et al. (2022), and 0.370–0.500 in the cereal bars developed by Samakradhamrongthai et al. (2021). In contrast, our samples contained added water to reduce the hardness of the ball and make it easier to chew, as the commercial product was hard and dry. However, the sugar content (10.64–11.26 g 100 g^−1^) and the amount of honey added (10.41%–12.51%) in our samples were considered low compared to other products. Typically, cereal bars use honey and sugar syrup as binding agents without added water, which results in high sugar content (Samakradhamrongthai et al., 2021). For example, in Samakradhamrongthai et al. (2021), corn syrup and honey (20%–26%) were used as sweeteners, and the sugar content ranged from 10.13 to 38.19 g 100 g^−1^. The commercial product had a sugar content of 35 g 100 g^−1^. However, our study did not aim to develop a product with high sugar content. According to Maleki et al. (2023), calories from protein and fat are preferred in protein snacks. The added water and reduced sugar content in our samples led to higher levels of water activity, which requires careful consideration of the packaging type, use of preservatives, and/or storage temperature to maintain a longer shelf‐life of the product.

### 4.2 Color

The color of a product is generally considered the most important attribute when it comes to the appearance of food (Akullo et al., 2023; MacDougall, 2001). In this study, the replacement of milk protein with cricket flour significantly decreased the lightness values of the balls both internally (Figure 2) and externally (Figure 3). This outcome was expected since milk protein powder is white, while cricket flour has a darker color. Similar observations were reported by González et al. (2019) and Ho et al. (2022) when cricket flour was added to their products, and by Sriprablom et al. (2022) when adding mealworm powder. The use of cricket flour also led to a decrease in the chroma (C*) values, particularly on the outer surface of the ball. The brown color of the samples with milk protein was more vibrant, whereas the cricket samples were duller. Regarding the Hue angle, it was generally similar for all formulations, which was in the direction of the yellow color matrix. However, there was a differentiation between the Hue angles of carob and cocoa formulations on the inside of the balls, as the color matrix of cocoa and carob powder differs slightly. The Hue angle of the carob powder (62.80° ± 0.47) was significantly higher than that of cocoa powder (56.63° ± 0.44).

Despite using cocoa and carob powder to mask the color of cricket flour, the color of the milk formulations and cricket formulations were noticeably different (Δ*E* > 5) according to Mokrzycki and Tatol (2011). The mean color difference, as measured by Δ*E*, between milk cocoa and cricket cocoa formulations was 7.0 externally and 10.9 internally, while it was 5.6 externally and 9.5 internally between milk carob and cricket carob.

Concerning the color change over time, for all formulations except for cricket cocoa, the change in color after 2 weeks of storage was minimal and likely unnoticeable to consumers (Δ*E* < 2). For the cricket cocoa formulation, the color difference was noticeable but not very clear, falling within the range of 2 < Δ*E* < 3.5 (Mokrzycki & Tatol, 2011). Therefore, the color of the balls was generally maintained when stored in the refrigerator for 2 weeks.

### 4.3 Texture profile analysis

Food texture is a complex characteristic that is directly linked to consumers´ sensory perception. Initially, the results showed no distinct pattern of difference between cricket flour and milk protein formulations, particularly in hardness and springiness. This indicates that replacing milk protein powder with cricket flour does not significantly affect the initial texture of the protein balls. However, after 2 weeks of refrigerated storage, the differences were clear in hardness and chewiness, with milk protein formulations displaying higher values in both parameters. The interactions between ingredients and the formation of bonds reveal the changes in texture over time. It appears that the cricket protein ball formulations are relatively more stable over time, maintaining their hardness without a significant increase. On the other hand, chewiness and springiness increased significantly for all four formulations, likely due to the absorption of moisture by the oat flakes and the formation of a gel‐like structure. This gel‐like structure may enhance the product's ability to spring back after being deformed.

The increase in cohesiveness in milk formulations over time, as opposed to the cricket formulations, can be attributed to the nature of the protein matrix (Delikanli & Ozcan, 2017). Milk protein, consisting of casein and whey, likely played a more significant role in maintaining the structural integrity and internal cohesion of the protein balls compared to cricket protein. Chitin, an insoluble fiber in cricket flour, contains non‐protein nitrogen, which can interfere with protein interactions, as Luna et al. (2021) explain. Similarly, Chailangka et al. (2023) found that replacing part of casein with cricket protein decreased the cohesiveness of mozzarella cheese.

The increased hardness in milk formulations after 2 weeks of storage, contrary to the cricket formulations, may be due to moisture migration, where proteins form bonds with sugars, resulting in a stronger network (Mihaylova et al., 2022). Milk protein was more likely to form these networks, thereby delaying the increase in hardness seen in cricket samples. Additionally, the slightly higher fat content in cricket formulations may have contributed to their lower hardness compared to the milk formulations. Since chewiness is a product of hardness, springiness, and cohesiveness, the increased hardness in the milk formulations, contributed to a higher increase in chewiness.

### 4.4 Microbiological analysis

The evaluation of mesophilic aerobes, yeasts, and molds can serve as an indicator of the organoleptic quality and shelf‐life of the product (HPA, 2009). On one hand, our product is categorized as a raw, ready‐to‐eat food product that has not been baked and is directly consumed without further cooking or processing. This explains the observed mesophilic aerobes count in all formulations (Figure 4) when compared to baked products, which typically have a mesophilic aerobes count below 3 log CFU g^−1^ due to the heat process involved (HPA, 2009). The Spanish norm of ready‐to‐eat food (BOE, 2001) states that the acceptable limit of the mesophilic aerobes count for food that has not undergone heat treatment is having two out of five samples with a count between 5 log and 6 log CFU g^−1^. Thus, our product was within the limit. Raw, ready‐to‐eat food such as vegetables in salads have mesophilic aerobic counts of 6 log to 8 log CFU g^−1^ (HPA, 2009), while Badosa et al. (2008) found that it was in the range of 7 log to 9 log CFU g^−1^ for packed ready‐to‐eat vegetables. In addition, raw meat and fish eaten untreated or cold smoked have mesophilic aerobic counts of around 6 log to 7 log CFU g^−1^ (HPA, 2009). Moreover, the water activity (0.9 on average) of our product supports microbial growth.

On the other hand, the results showed that replacing milk protein powder with cricket flour has significantly increased the mesophilic aerobes count (Figure 4). Microbiological analysis of the raw materials confirmed the higher microbial load found in cricket flour compared to milk protein isolate. Furthermore, Fernandez‐Cassi et al. (2018) explain that the mesophilic aerobes count in cricket is high since the whole animal is processed, including the gut. Kooh et al. (2020) also explain that when mealworm (*Tenebrio molitor*) powder is rehydrated, microbes can easily multiply. Therefore, there should be recommendations for storage time and temperature of insect‐based products of high water activity like, for example, protein shakes (*a*_w_ = 0.99). In our case, the non‐baked protein balls (*a*_w_ = 0.9) were stored in a refrigerator (2°C ± 1°C) and the count was maintained after 2 weeks of storage (Figure 4). Compared to our product, the commercial protein ball had lower counts of mesophilic aerobes and yeasts and molds due to modified atmosphere packaging, lower water activity, reduced moisture content, and the use of a different protein source (whey protein isolate).

As for the yeasts and mold count, the values varied across different formulations without a clear effect from the main protein source. According to HPA (2009), yeasts may cause spoilage at levels of 10^6^–10^7^ CFU g^−1^ due to acid and gas production. Hence, our results (Figure 5) indicate that the refrigerated storage of the different protein ball formulations for 2 weeks did not lead to spoilage due to yeast growth.

### 4.5 Descriptive sensory analysis

The sensory profile of the five different protein ball samples was obtained using GPA followed by principal component analysis (PCA). This method has determined the sensory space of the five different protein balls and identified key sensory descriptors, confirming the rapidity and reliability of the method. The residuals by sample after transformations were the lowest for the milk cocoa sample, suggesting high agreement among panelists in sensory evaluation, likely due to their familiarity with milk protein and cocoa flavors. The elimination of attributes in our case increased the variability explained by the first two components from 84.61% to 95.14%, but reduced the consensus index (*R*_C_) from 0.602 to 0.371. This allowed describing the samples more meaningfully, focusing on the main attributes that define each sample.

The sensory analysis allowed obtaining a comprehensive overview of the primary sensory attributes of the different protein ball formulations, adding value to the instrumental measurements. For example, it highlighted the distinct flavors associated with the cricket samples, such as woody aroma, aroma intensity, odd taste, and flavor intensity. These characteristics stem from volatile organic compounds present in cricket flour (Ochieng et al., 2023), as perceived by some panelists. The aroma intensity could also be influenced by the potent flavor of roasted peanuts from peanut butter, potentially masking the cricket flavor. Some panelists also perceived an odd taste in the commercial sample, possibly due to concentrated ingredients in the product, such as dates, grape juice concentrate, or whey protein isolate. According to Liu et al. (2021), proteins impart specific flavors, such as astringency, which increases with the increase in protein content. Additionally, a greasy and shiny appearance was perceived by some of the panelists, predominantly in the commercial sample, which may be due to the visible peanut pieces on its surface, unlike the other samples made with peanut butter. These attributes are associated with the product's fat content and the oily texture.

The descriptive sensory analysis also complements some of the parameters measured by the instruments. For example, the panel confirmed the increased lightness in the color of the milk formulations, as measured by the colorimeter (Figures 2 and 3) compared to the cricket formulations. Moreover, as measured by the texture analyzer and interpreted by the sensory analysis, the milk and cricket samples had a low level of chewiness (Table 4) where they tended toward the attribute tenderness compared to the commercial product. The low moisture content of the commercial product (Appendix S3) was also perceived by the panel in the sensory analysis as they associated it with dryness.

## 5 CONCLUSION

Our study demonstrated a potential application of cricket flour in protein balls without adverse impacts on organoleptic properties. The cricket flour was suitable at levels (7%) that can be utilized to develop a product with a “high protein” claim for consumers intending to increase the protein intake in their diet. Compared to the milk protein formulations, cricket flour had minor effects on color. It had a positive effect on texture with maintained hardness after 2 weeks of storage. As for the microbiological quality, it was within the limit for 2 weeks of storage without a significant change. Carob powder was also demonstrated to be a suitable alternative to cocoa at a 3% addition level without noticeable effects on the properties studied. Compared to the commercial product, our product has a similar appearance and protein content, but with a reduced calorie content and a more desirable texture with reduced hardness and chewiness while compromising storage conditions. Further research is needed to study the stability of the product during longer periods of storage and to explore different packaging types (vacuum, modified atmosphere, etc.). A hedonic evaluation by potential consumers is also important to determine the acceptability of the product.

## AUTHOR CONTRIBUTIONS

**Reine Khalil:** Conceptualization (equal); data curation (lead); formal analysis (lead); methodology (equal); visualization (equal); writing – original draft (lead); writing – review and editing (equal). **Zein Kallas:** Conceptualization (equal); funding acquisition (equal); methodology (equal); resources (equal); supervision (equal); validation (equal); writing – review and editing (equal). **Montserrat Pujolà:** Conceptualization (equal); methodology (equal); resources (equal); supervision (equal); validation (equal); writing – review and editing (equal). **Amira Haddarah:** Conceptualization (equal); methodology (equal); supervision (equal); validation (equal); writing – review and editing (equal).

## FUNDING INFORMATION

This study is part of the SUSPROMO project, grant PID2019‐111716RB‐I00, funded by the MCIN (Spanish Ministry of Science and Innovation)/AEI (Spanish Research Agency, DOI: 10.13039/501100011033). The content of this paper reflects only the author's view and the MCIN/AEI is not responsible for any use that may be made of the information it contains.

## CONFLICT OF INTEREST STATEMENT

The authors declare that they have no competing interests.

## ETHICS STATEMENT

Ethical approval was obtained from the ethics committee of the Center for Agro‐food Economics and Development (CREDA).

## INFORMED CONSENT

Written informed consent was obtained from all study participants.

## Supporting information


Appendix S1

## Data Availability

Data will be made available upon reasonable request.
