# Supplementary material for: Organoleptic characteristics of high‐protein snacks with novel and sustainable ingredients: Cricket flour and carob powder
Source: Food Sci Nutr. 2024 Oct 14;12(11):9443–57. doi: 10.1002/fsn3.4392 (PMC11606854; doi:10.1002/fsn3.4392)
Supplement: Supplementary file 1 — Appendix S1 [file FSN3-12-9443-s001.docx]

**Appendix 1**

**Flash Profile of Protein Balls**

**Assessor name:**

**Product Description**

The product to be evaluated is a protein ball developed for physically active people. It can be consumed as a convenient post-workout snack to aid in muscle recovery. The product is high in protein with 19-22 g of protein per 100 g product.

**Allergens:** peanuts, cricket powder (which is linked to allergies to crustaceans and dust mites), and milk protein. The oat flakes used are declared gluten-free on the package label.

**Evaluation Procedure Description**

In this first session, you will be ranking the samples based on the **attributes that best describe the differences among the samples**. Some attributes are already listed for you to simplify the attribute generation process. Nevertheless, you may generate more attributes and add them to the list or exclude the existing ones. It is possible to discuss the attributes with other assessors. You can use the sheet of attributes presented to you for assistance. Please **avoid the use of hedonic terms**.

Please sort the samples in **ascending order** for each attribute (from low to high intensity). If no difference is perceived, you may apply the same rank (ties) to two or more samples by underlining or circling the sample codes.

Please taste each sample individually, taking some toast and water between the samples. You are allowed to evaluate the samples in any order and you can re-taste them as many times as you want.

***N.B.:*** It is recommended to not have many attributes, preferably not more than 10, since the samples are saturating.

| Attributes | Attribute Description | Ascending order | | | | |
| --- | --- | --- | --- | --- | --- | --- |
| Appearance low intensity (-) high intensity (+) | | | | | | |
| Color intensity | Darkness |  |  |  |  |  |
|  |  |  |  |  |  |  |
|  |  |  |  |  |  |  |
| Aroma low intensity (-) high intensity (+) | | | | | | |
| Overall intensity | Intensity of aroma |  |  |  |  |  |
|  |  |  |  |  |  |  |
|  |  |  |  |  |  |  |
| Taste/After-taste low intensity (-) high intensity (+) | | | | | | |
| Sweetness | Basic taste elicited by sucrose |  |  |  |  |  |
| Odd taste | Distinguishable taste not perceived in all samples |  |  |  |  |  |
|  |  |  |  |  |  |  |
|  |  |  |  |  |  |  |
| Texture low intensity (-) high intensity (+) | | | | | | |
| Sandiness | Low level of granularity related to particle size perceived by the mouth |  |  |  |  |  |
| Dryness | Perception of moisture level by the mouth |  |  |  |  |  |
| Tenderness | Low level of chewiness, easy to swallow |  |  |  |  |  |
|  |  |  |  |  |  |  |
|  |  |  |  |  |  |  |
|  |  |  |  |  |  |  |

**Flash Profile of Protein Balls**

**Assessor name:**

**Product Description**

The product to be evaluated is a protein ball developed for physically active people. It can be consumed as a convenient post-workout snack to aid in muscle recovery. The product is high in protein with 19-22 g of protein per 100 g product.

**Allergens:** peanuts, cricket powder (which is linked to allergies to crustaceans and dust mites), and milk protein. The oat flakes used are declared gluten-free on the package label.

**Evaluation Procedure Description**

In this second session, you will be ranking the samples based on the **attribute list of the first session**.

Please sort the samples in **ascending order** for each attribute (from low to high intensity). If no difference is perceived, you may apply the same rank (ties) to two or more samples by underlining or circling the sample codes.

Please taste each sample individually, taking some toast and water between the samples. You are allowed to evaluate the samples in any order and you can re-taste them as many times as you want.

| Attributes | Attribute Description | Ascending order | | | | |
| --- | --- | --- | --- | --- | --- | --- |
| Appearance low intensity (-) high intensity (+) | | | | | | |
|  |  |  |  |  |  |  |
|  |  |  |  |  |  |  |
|  |  |  |  |  |  |  |
| Aroma low intensity (-) high intensity (+) | | | | | | |
|  |  |  |  |  |  |  |
|  |  |  |  |  |  |  |
|  |  |  |  |  |  |  |
| Taste/After-taste low intensity (-) high intensity (+) | | | | | | |
|  |  |  |  |  |  |  |
|  |  |  |  |  |  |  |
|  |  |  |  |  |  |  |
|  |  |  |  |  |  |  |
| Texture low intensity (-) high intensity (+) | | | | | | |
|  |  |  |  |  |  |  |
|  |  |  |  |  |  |  |
|  |  |  |  |  |  |  |
|  |  |  |  |  |  |  |
|  |  |  |  |  |  |  |
|  |  |  |  |  |  |  |

**Appendix 2**

**Table S1.** P values of Kruskal-Wallis test and Spearman correlation coefficients for panelists’ attribute evaluations by Flash Profile

| **Panelist 1** | **Attribute** | **Kruskal Wallis**  **P value** | **Spearman correlation coefficient** |
| --- | --- | --- | --- |
|  | color intensity | 0.073 | 0.900** |
|  | aroma intensity | 0.147 | 0.462 |
|  | strange aroma | 0.170 | 0.791 |
|  | sweetness | 0.074 | 0.921** |
|  | odd taste | 0.170 | 0.791 |
|  | bitterness | 0.061 | 1.000*** |
|  | sandiness | 0.061 | 1.000*** |
|  | dryness | 0.399 | -0.100 |
|  | tenderness | 0.073 | 0.900** |

| **Panelist 2** | **Attribute** | **Kruskal Wallis**  **P value** | **Spearman correlation coefficient** |
| --- | --- | --- | --- |
|  | color intensity | 0.073 | 0.900** |
|  | aroma intensity | 0.260 | 0.154 |
|  | sweetness | 0.998 | -0.975*** |
|  | odd taste | 0.140 | 0.649 |
|  | sandiness | 0.147 | 0.462 |
|  | dryness | 0.329 | 0.051 |
|  | tenderness | 0.406 | 0.800 |
|  | stickiness | 0.406 | NA |

| **Panelist 3** | | **Attribute** | **Kruskal Wallis**  **P value** | | **Spearman correlation coefficient** |
| --- | --- | --- | --- | --- | --- |
|  | | color intensity | 0.073 | | 0.900** |
|  | | aroma intensity | 0.343 | | 0.000 |
|  | | sweetness | 0.772 | | -0.600 |
|  | | odd taste | 0.105 | | 0.700 |
|  | | sandiness | 0.075 | | 0.949** |
|  | | dryness | 0.150 | | 0.500 |
|  | | tenderness | 0.061 | | 1.000*** |
|  | | hardness | 0.105 | | 0.900* |
|  | | gumminess | 1.000 | | -1.000*** |
| **Panelist 4** | **Attribute** | | | **Kruskal Wallis**  **P value** | **Spearman correlation coefficient** |
|  | color intensity | | | 0.061 | 1.000*** |
|  | peanut aroma intensity | | | 0.073 | 0.900** |
|  | sweetness | | | 0.245 | 0.158 |
|  | bitterness | | | 0.609 | -0.400 |
|  | sandiness | | | 0.211 | 0.300 |
|  | dryness | | | 0.343 | 0.000 |
|  | tenderness | | | 0.292 | 0.100 |

| **Panelist 5** | **Attribute** | **Kruskal Wallis**  **P value** | **Spearman correlation coefficient** |
| --- | --- | --- | --- |
|  | color intensity | 0.073 | 0.900** |
|  | shiny appearance | 0.099 | 0.825* |
|  | aroma intensity | 0.147 | 0.462 |
|  | sweetness | 0.270 | 0.216 |
|  | sandiness | 0.129 | 0.718 |
|  | dryness | 0.061 | 1.000*** |
|  | tenderness | 0.061 | 1.000*** |
|  | stickiness | 0.150 | 0.500 |

| **Panelist 6** | **Attribute** | **Kruskal Wallis**  **P value** | **Spearman correlation coefficient** |
| --- | --- | --- | --- |
|  | color intensity | 0.465 | -0.108 |
|  | homogeneity | 0.273 | 0.289 |
|  | aroma intensity | 0.406 | NA |
|  | sweetness | 0.718 | -0.462 |
|  | odd taste | 0.524 | -0.224 |
|  | dryness | 0.635 | -0.316 |
|  | tenderness | 0.406 | NA |
|  | hardness | 0.126 | 0.600 |
|  | granularity | 0.140 | 0.649 |

| **Panelist 7** | **Attribute** | **Kruskal Wallis**  **P value** | **Spearman correlation coefficient** |
| --- | --- | --- | --- |
|  | color intensity | 0.105 | 0.700 |
|  | presence of black particles | 0.061 | 1.000*** |
|  | aroma intensity | 0.211 | 0.300 |
|  | woody aroma intensity | 0.061 | 1.000*** |
|  | peanut aroma intensity | 0.343 | 0.000 |
|  | sweetness | 0.150 | 0.500 |
|  | odd taste | 0.075 | 0.949** |
|  | peanut taste intensity | 0.406 | 0.975*** |
|  | sandiness | 0.145 | 0.707 |
|  | dryness | 0.145 | 0.707 |
|  | tenderness | 0.145 | 0.707 |

| **Panelist 8** | **Attribute** | **Kruskal Wallis**  **P value** | **Spearman correlation coefficient** |
| --- | --- | --- | --- |
|  | color intensity | 0.068 | 0.973*** |
|  | aroma intensity | 0.216 | 0.359 |
|  | sweetness | 0.830 | -0.667 |
|  | odd taste | 0.068 | 0.973*** |
|  | stickiness | 0.061 | 1.000*** |
|  | hardness | 0.170 | 0.791 |
|  | dryness | 0.145 | 0.707 |

| **Panelist 9** | **Attribute** | **Kruskal Wallis**  **P value** | **Spearman correlation coefficient** |
| --- | --- | --- | --- |
|  | color intensity | 0.105 | 0.700 |
|  | aroma intensity | 0.190 | 0.287 |
|  | sweetness | 0.525 | -0.289 |
|  | odd taste | 0.588 | -0.395 |
|  | sandiness | 0.170 | 0.791 |
|  | dryness | 0.170 | 0.791 |
|  | tenderness | 1.000 | -1.000*** |
|  | hardness | 0.170 | 0.791 |

| **Panelist 10** | **Attribute** | **Kruskal Wallis**  **P value** | **Spearman correlation coefficient** |
| --- | --- | --- | --- |
|  | color intensity | 0.073 | 0.900** |
|  | greasy appearance | 0.083 | 0.821* |
|  | aroma intensity | 0.150 | 0.500 |
|  | sweetness | 0.399 | -0.100 |
|  | odd taste | 0.406 | NA |
|  | bitterness | 0.406 | NA |
|  | sandiness | 0.061 | 1.000*** |
|  | dryness | 0.061 | 1.000*** |
|  | tenderness | 0.061 | 1.000*** |

| **Panelist 11** | **Attribute** | **Kruskal Wallis**  **P value** | **Spearman correlation coefficient** |
| --- | --- | --- | --- |
|  | color intensity | 0.061 | 1.000*** |
|  | shiny appearance | 0.150 | 0.500 |
|  | aroma intensity | 0.437 | -0.264 |
|  | peanut aroma intensity | 0.265 | 0.205 |
|  | odd taste | 0.061 | 1.000*** |
|  | flavor intensity | 0.073 | 0.900** |
|  | sandiness | 0.810 | -0.667 |
|  | fattiness | 0.718 | -0.462 |
|  | stickiness | 0.136 | 0.556 |

(*: significance at 0.1 level, **: significance at 0.05 level, ***: significance at 0.01 level)

**Appendix 3**

**Table S2.** Description of the characteristics of the commercial protein ball

| **Parameter** | **Value** |
| --- | --- |
| Water activity | 0.599±0.003 |
| Moisture (g 100 g^-1^) | 17.76±0.55 |
| **Nutritional Data (per 100g)** | |
| Fat (g) | 20 |
| Protein (g) | 22 |
| Carbohydrates (g) | 38 |
| Sugar (g) | 35 |
| Fiber (g) | 6 |
| Calories (Kcal) | 434 |
| **Color (Internal)** | |
| Lightness | 31.82±2.45 |
| Chroma | 12.68±1.10 |
| Hue (^o^) | 54.15±3.72 |
| **Color (External)** |  |
| Lightness | 30.38±2.71 |
| Chroma | 11.35±0.81 |
| Hue (^o^) | 49.38±5.89 |
| **Texture Profile Analysis** |  |
| Hardness (N) | 226.78±17.63 |
| Springiness (mm) | 0.29±0.02 |
| Cohesiveness (-) | 0.22±0.01 |
| Chewiness (N.mm) | 13.86±1.13 |
| **Microbiological Analysis** |  |
| Mesophilic aerobes (log CFU g^-1^) | 2.38±0.14 |
| Yeasts and Molds (log CFU g^-1^) | 1.26±0.00 |

Values represent mean ± standard deviation except for nutritional data.

Sample size: n=3 for water activity, moisture, and color; n= 12 for texture profile analysis (load cell 50 kg)
